# The Influence of Basic Therapy and New Drugs on NO-Dependent Mechanisms of Cardiac Destruction in Chronic Heart Failure

**DOI:** 10.3390/biomedicines14051018

**Published:** 2026-04-30

**Authors:** Igor Belenichev, Olena Popazova, Olexiy Goncharov, Nina Bukhtiyarova, Victor Ryzhenko, Denys Semenov, Sergiy Oliynyk, Pavlo Petakh, Oleksandr Kamyshnyi

**Affiliations:** 1 Department of Pharmacology and Medical Formulation with Course of Normal Physiology, Zaporizhzhia State Medical and Pharmaceutical University, 69000 Zaporizhzhia, Ukraine or belenichev.i.f@zsmu.edu.ua (I.B.); goncharov.ov@zsmu.edu.ua (O.G.); 2 Department of Histology, Cytology and Embryology, Zaporizhzhia State Medical and Pharmaceutical University, 69000 Zaporizhzhia, Ukraine; 3 Department of Clinical Laboratory Diagnostics, Zaporizhzhia State Medical and Pharmaceutical University, 69000 Zaporizhzhia, Ukraine; nvb21nm@gmail.com; 4 Department of Medical and Pharmaceutical Informatics and Advanced Technologies, Zaporizhzhia State Medical and Pharmaceutical University, 69000 Zaporizhzhia, Ukraine; ryzhenko.victor@gmail.com; 5 Department of Propaedeutic Dentistry and Oral Surgery, Zaporizhzhia State Medical and Pharmaceutical University, 69000 Zaporizhzhia, Ukraine; emdenmix3@gmail.com; 6 School of Medicine, Ewha Womans University, 52, Ewhayeodaegil, Seodaemun-gu, Seoul 03760, Republic of Korea; sergiyoliynyk622@gmail.com; 7 Department of Biochemistry and Pharmacology, Uzhhorod National University, 88000 Uzhhorod, Ukraine; 8 Department of Microbiology, Virology and Immunology, I. Horbachevsky Ternopil State Medical University, 46001 Ternopil, Ukraine; alexkamyshnyi@gmail.com

**Keywords:** chronic heart failure, nitric oxide, mitochondrial dysfunction, reactive oxygen species, oxidative stress, apoptosis, β-blockers, ACE inhibitors

## Abstract

Chronic heart failure (CHF) remains a leading cause of global mortality, characterized by profound molecular and biochemical disturbances, including nitric oxide (NO) system dysfunction, mitochondrial impairment, and oxidative stress. While standard therapies such as ACE inhibitors, SGLT2 inhibitors, and beta-blockers address clinical symptoms, their capacity to interrupt the underlying biochemical mechanisms of cardiomyopathy is often limited. This review examines the pathophysiological role of impaired NO production and reactive oxygen species (ROS) accumulation in exacerbating myocardial contractile dysfunction and disease progression. Special focus is directed toward the development of next-generation β1-blockers with multifunctional properties, including antioxidant, NO-mimetic, and antiapoptotic effects. Evidence suggests that the novel compound Hypertril (1-(β-phenylethyl)-4-amino-1,2,4-triazolium bromide) exhibits significant cardioprotective potential. Experimental data indicate that Hypertril improves eNOS/iNOS expression and enhances NO bioavailability more effectively than conventional β-blockers, leading to stabilized ECG parameters and restored energy metabolism. These findings underscore the clinical relevance of developing NO-mimetic agents to optimize the pharmacological management of CHF.

## 1. Introduction

Heart failure is currently one of the leading causes of mortality in most countries worldwide. It affects 0.5–2% of the adult population; however, among individuals over 65 years of age, the prevalence rises to 6–10% [1,2,3]. Despite significant advances in the treatment of cardiovascular diseases, the prevalence of chronic heart failure (CHF) not only remains high but continues to increase steadily. According to various reports, the annual incidence reaches approximately 300 cases per 100,000 population. Depending on disease severity, mortality rates in CHF vary considerably, ranging from 15% to 50% [4,5,6]. More than 40% of patients with signs of CHF present with terminal, class III-IV functional classification of the disease. In 2023, decompensated CHF was the cause of hospitalization in cardiology departments for nearly every second patient (49%), and CHF was listed in the diagnosis of 92% of those admitted to such facilities. The one-year mortality rate for patients with clinically manifest CHF reaches 26–29% [7]. The main causes of CHF are myocardial infarction, hypertension, cardiomyopathy, and valvular heart diseases [8,9]. After myocardial infarction, the heart usually adapts through a pathophysiological process known as cardiac remodeling, which involves alterations in the structure and function of cardiomyocytes as well as in the extracellular matrix of the non-infarcted myocardium. These changes result in significant modifications of cardiac shape and volume, progressive ventricular dilatation, and impaired contractile function [10]. The analysis of the mechanisms underlying disorders in CHF remains an exceptionally complex task. They have proven to be much broader, more diverse, and more intricate than previously assumed [11,12]. Therefore, the investigation of the molecular mechanisms of cardiomyocyte injury in CHF, the identification of potential pharmacological targets, and the development of novel cardioprotective agents represent a crucial task for modern medicine and pharmacy. Based on the above, we set the goal of generalizing and structuring the interconnected NO-dependent mechanisms of cardiac destruction in CHF based on the analysis of the results of fundamental and clinical studies, as well as evidence of the interruption of these mechanisms in basic heart failure therapy and new potential drugs. Based on an analysis of open sources and the results of our own research, this paper summarizes and systematizes data on the leading molecular and biochemical mechanisms of cardiodestruction (nitric oxide system disturbances, apoptosis, oxidative stress, substrate alterations, energy deficit, and mitochondrial dysfunction) in CHF. Furthermore, it highlights the role of NO-dependent cardioprotective mechanisms of both standard medications and novel original compounds.

## 2. Molecular and Biochemical Mechanisms of Myocardial Dystrophy in CHF—Focus on Nitric Oxide (NO)

**Figure 1:**
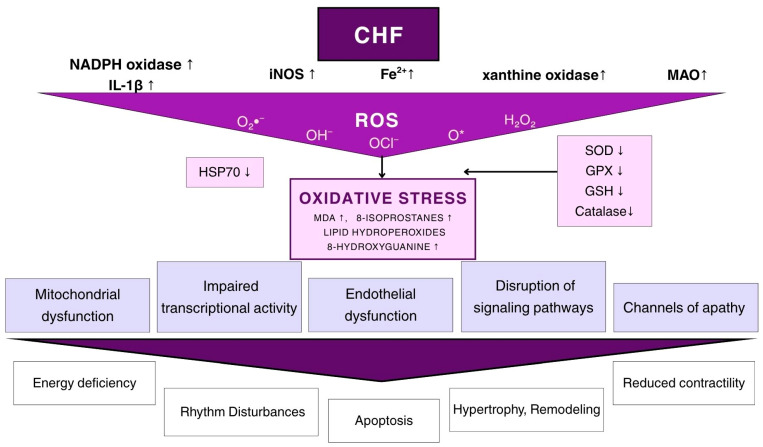
Cascade of Molecular and Biochemical Reactions Leading to Cardiac Destruction in Chronic Heart Failure.

The development of heart failure involves multiple mechanisms. One of the most critical links in CHF pathogenesis is oxidative stress, as evidenced by damage induced by reactive oxygen species (ROS), including lipid peroxidation, as well as a decrease in antioxidant levels and sulfhydryl groups. Damage to myofibrils and impaired regulation of intracellular calcium under the influence of ROS are also key mechanisms commonly associated with the pathogenesis of CHF [13,14]. The targets of apoptosis in CHF are not limited to cardiomyocytes but also include endothelial cells, as indicated by caspase activation and internucleosomal DNA degradation [15]. In the pathogenesis of CHF, additional mechanisms have been identified, such as alterations in the high-energy phosphate pool and the development of secondary mitochondrial dysfunction against the background of energy deficiency [16]. All these molecular mechanisms of molecular and biochemical disturbances are closely associated with alterations in the myocardial nitroxidergic system (Figure 1).

Multiple mechanisms are involved in cardiac destruction in CHF, but oxidative stress is the most studied pathogenesis. This is evidenced by functional and structural damage caused by lipid peroxidation, oxidative modification of proteins and nucleic acids, and reactive oxygen species (ROS) triggered by deprivation of the antioxidant system. The following ROS play a role in the pathogenesis of CHF: superoxide radical (O_2_^−^), hydroxyl radical (OH^−^), hydrogen peroxide (H_2_O_2_), singlet oxygen (O*_2_), ozone (O_2_), hypochlorite anion (OCl^−^), peroxyl radicals (ROO_2_), alkoxyl radicals (RO_2_), as well as active forms of nitric oxide, which are formed from NO during its increased production under the influence of ROS: peroxynitrite (ONOO^−^), nitrosonium cation (NO^+^), nitroxide anion (NO^−^) and S-nitrosothiols. In CHF, the main pathways for the formation of nitric oxide ROS are: discoordination of mitochondrial electrotransport chains against the background of increased expression of NAD(P)H oxidase in secondary mitochondrial dysfunction, decreased pO_2_ in the myocardium, accumulation of reduced forms of pyridine nucleotides; accumulation of catecholamines, their precursors and metabolic products, activation of adenyl nucleotide metabolism and increased expression of xanthine oxidase, imbalance of trace elements in the myocardium (Fe, Cu, Zn, Mn, etc.), increased arachidonic acid metabolism, decreased expression of antioxidant enzymes (superoxide dismutase, catalase, glutathione peroxidase, glutathione reductase) and deficiency of endogenous antioxidants (a-tocopherol, glutathione, cysteine, etc.), increased production of NO under the influence of iNOS. Uncontrolled production of ROS and NO leads to oxidative stress, resulting in increased formation of cytotoxic and cardiotoxic products (MDA, 8-epi-isoprostanes, 8-hydroxyguanine, 4-hydroxy-trans-2-nonenal, carbonyl derivatives of proteins (aldehydes and ketones)). Oxidative stress causes changes in intracellular pathways, in Red/Oxi-signaling, aggravates secondary mitochondrial dysfunction, causes endothelial dysfunction, receptor desensitization and secondary channelopathies, and subsequently leads to myocardial hypertrophy, damage to the functional activity of the heart, apoptosis of cardiomyocytes, increased maladaptive remodeling and causes the progression of CHF.

### 2.1. Mitochondrial Dysfunction and Impairments of Energy Metabolism in CHF

Mitochondria represent the most extensively and progressively damaged subcellular organelles in cardiac pathology, including CHF [17,18,19,20]. In CHF, energy metabolism is impaired, characterized by reduced ATP production due to mitochondrial dysfunction and a shift from efficient glucose oxidation to less efficient fatty acid oxidation. Cardiomyocytes experience an energy deficit, a significant decrease in ATP required for cardiac contractile activity, which leads to diminished cardiac function and efficiency. It is highly likely that the energy deficit in CHF is associated with the development of secondary mitochondrial dysfunction. Mitochondrial dysfunction in CHF causes changes in substrate preference and alterations in transcriptional and signaling pathways, thereby triggering apoptosis, inflammation, and ROS generation [21,22,23,24,25]. These disturbances lead to ultrastructural pathological changes, such as mitochondrial swelling and the formation of myelin figures within mitochondria [26]. In a doxorubicin-induced CHF model in rats, it was found that the oxidation of long-chain fatty acids in cardiac mitochondria was significantly reduced, whereas glucose metabolism was increased, indicating an overall shift from an aerobic to an anaerobic metabolic state [27].

Such changes in both glycolysis and mitochondrial oxidative metabolism in CHF are driven not only by transcriptional alterations of key enzymes involved in these metabolic pathways but also by modifications in the redox state of NAD^+^/NADH and metabolite signaling, which promote post-translational epigenetic changes in the regulation of genes encoding energy metabolism enzymes. Alterations in glucose fate, beyond its flux through glycolysis or glucose oxidation, also contribute to the pathology of CHF [28].

**Figure 2:**
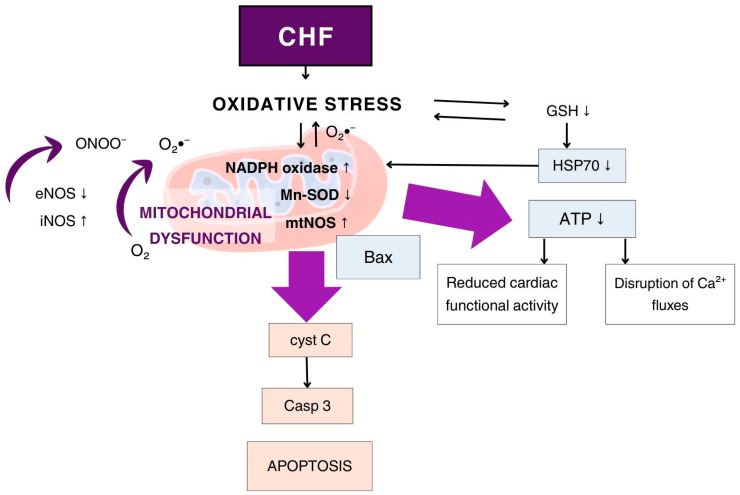
The Role of Mitochondrial Dysfunction in Myocardial Injury in Chronic Heart Failure.

This described metabolic shift is characteristic of CHF and can lead to significant lactate accumulation and lactic acidosis. The role of NO and various NOS isoforms in the development of mitochondrial dysfunction in CHF is also well established. Excessive NO production, in the context of increased iNOS expression and thiol antioxidant deficiency, leads to damage of the mitochondrial electron transport chain and may contribute to the development of mitochondrial dysfunction in CHF (Figure 2) [29,30,31].

It has been shown that in CHF, mitochondria represent an important source of NO and its cytotoxic derivatives. Mitochondrial NOS is significantly activated in response to acute and chronic myocardial ischemia, mitochondrial calcium uptake, increased superoxide radical levels, as well as in response to inflammation and elevated concentrations of IL-1β and TNF-α. Mitochondrial production of NO and superoxide radicals leads to their interaction and formation of a highly reactive species—peroxynitrite, which in turn triggers the opening of the mitochondrial permeability transition pore (mPTP) [32,33]. Peroxynitrite (ONOO^−^) also nitrosylates cytochrome C in mitochondria, leading to altered function; in particular, it becomes incapable of supporting electron transfer in the respiratory chain [34]. Additionally, peroxynitrite (ONOO^−^), through nitrosylation of active sites in mitochondrial membrane proteins, negatively affects calcium homeostasis in cardiomyocytes during CHF and myocardial ischemia [32,35]. Mitochondria are known to play a key role in the regulation of intracellular calcium, which is essential for the excitation–contraction coupling of cardiomyocytes.

In CHF, the myocardium exhibits a decreased density of intact mitochondria and an increased density of mitochondria with pronounced ultrastructural abnormalities, including enlarged size, fragmented outer membranes, disrupted cristae, electron-lucent matrix, cardiolipin deficiency in the inner membrane, and cristae remodeling [36].

Mitochondrial dysfunction also leads to suppression of the expression of numerous enzymes, such as succinate dehydrogenase, malate dehydrogenase, and superoxide dismutase, as well as protective proteins, including HSP70 and HIF-1α [37,38]. Persistent mitochondrial dysfunction initiates mechanisms of both extracellular and intracellular apoptosis. This occurs through the irreversible opening of the mPTP and the release of large amounts of pro-apoptotic factors into the cytosol. The opening of the mPTP is induced by NO and its reactive derivatives—peroxynitrite and the nitrosonium ion—which nitrosylate and subsequently oxidize the sulfhydryl (–SH) groups of cysteine residues in mitochondrial inner membrane antiporter proteins, leading to increased polarity and permeability for ions and proteins [39,40].

### 2.2. Impaired Delivery of Oxidative Substrates in the Myocardium in CHF

In heart failure, cardiomyocytes exhibit metabolic inflexibility, characterized by reduced fatty acid oxidation and increased glycolysis. This shift decreases ATP output and elevates ROS levels due to Krebs cycle dysregulation and glycolysis activation [41]. Mitochondria in endothelial cells during CHF begin to utilize increased amounts of fatty acids, which further enhances ROS production, reduces NO generation due to decreased eNOS expression, and exacerbates mitochondrial dysfunction [42]. Under normal conditions, NO regulates the transport and utilization of fatty acids in the myocardium by affecting their transport across the sarcolemma via FAT/CD36, influencing both fatty acid uptake and subsequent mitochondrial β-oxidation. In myocardial ischemia and CHF, dysregulation of the NO system, characterized by reduced bioavailability and elevated concentrations of reactive NO species, can lead to excessive fatty acid accumulation and lipotoxicity in the myocardium [43]. In the blood of patients with CHF, both with preserved and reduced ejection fraction, as well as in experimental models of CHF, elevated concentrations of ketone bodies have been observed. Ketone bodies are considered evolutionarily conserved fuels for myocardial cellular metabolism, intended to supply energy during periods of ischemia and substrate deprivation, and their increase is generally regarded as adaptive. However, the question of the adaptiveness of ketogenesis in CHF remains under investigation [44].

Increased ketone body oxidation is accompanied by a parallel upregulation of ketolytic enzymes, β-hydroxybutyrate dehydrogenase-1 (BDH1) and succinyl-CoA:3-oxoacid CoA transferase (SCOT), in the heart. Insufficient expression of these enzymes, particularly SCOT, may be associated with increased severity of CHF [45]. This occurs due to increased iNOS production in CHF. NO deficiency leads to reduced SCOT expression and a marked decline in myocardial energy capacity during CHF [36]. Impaired delivery of oxidative substrates, energy depletion, oxidative stress, and cardiomyocyte apoptosis in CHF primarily result from mitochondrial dysfunction [37].

## 3. Dysregulation of the Nitric Oxide System in CHF

In CHF, iNOS plays a significant role, as lactic acidosis can activate its expression in endothelial and vascular smooth muscle cells, leading to NO overproduction. Excess NO induces vasodilation, further exacerbating the condition by reducing vascular tone and arterial pressure, which can impair cardiac contractility and increase the risk of arrhythmias and hypotension [46]. In CHF, eNOS expression is reduced, leading to decreased NO production and contributing to endothelial dysfunction, impaired cardiac conduction, and diminished contractility. Reduced eNOS expression results in decreased vasodilation, increased oxidative stress, and heightened inflammation, which further impair vascular function and promote the progression of heart failure. In the myocardial microcirculation of patients with heart failure, decreased eNOS expression and reduced basal NO release have been observed [47,48]. Left ventricular dysfunction leads to reduced stroke volume, which in turn diminishes shear stress.

### 3.1. Reactive Oxygen Species and Energy Metabolism in the Myocardium

There is evidence for the impact of ROS on PGC-1α and PGC-1β—transcriptional coactivator proteins that play a key role in the regulation of energy metabolism, mitochondrial function, and mitochondrial biogenesis under both physiological conditions and cardiovascular pathology. PGC-1α and PGC-1β are partially functionally redundant, but both are required for normal development and proper functioning of the organism, particularly the cardiovascular system and skeletal muscles. PGC-1α mediates adaptive changes in physical activity patterns through the myocyte enhancer factor 2 and HIF-1α under hypoxic conditions [49]. The expression and promoter activity of PGC-1α and PGC-1β are increased in response to ROS, leading to altered expression of genes involved in mitochondrial electron transport as well as mitochondrial and cytosolic energy production systems. Different ROS concentrations exert distinct effects on PGC-1α and PGC-1β expression: levels slightly above physiological promote their expression and enhance mitochondrial biogenesis during cardiac hypertrophy, whereas high ROS concentrations suppress these factors, contributing to energy deficit and mitochondrial dysfunction [50,51]. Furthermore, varying ROS levels generated by NADPH oxidase 4 (NOX4) in cardiomyocytes can modulate myocardial angiogenesis under pressure-overload-induced stress [52].

### 3.2. ROS and Heart Failure

Excessive production of ROS by damaged mitochondria in CHF suppresses antioxidant defenses, leading to oxidative damage. Elevated levels of stable oxidative stress markers have been shown to positively correlate with worsening cardiac hemodynamic parameters in CHF. ROS-induced signaling contributes to cardiac remodeling by regulating the coordinated enlargement of cardiomyocytes, mitochondrial biogenesis, and capillary density during CHF. Moreover, in CHF, ROS modulate apoptotic signaling pathways [53]. Currently known ROS include: superoxide radical (O_2_^−^), hydroxyl radical (•OH), hydrogen peroxide (H_2_O_2_), singlet oxygen (^1^O_2_), ozone (O_3_), hypochlorite anion (OCl^−^), peroxyl radicals (ROO•), and alkoxyl radicals (RO•). In addition, reactive nitrogen species (RNS), which are derived from NO, include peroxynitrite (ONOO^−^), nitrosonium cation (NO^+^), nitroxide anion (NO^−^), and S-nitrosothiols. These reactive nitrogen species are generated as a result of oxidative modification under the influence of ROS when the antioxidant system is impaired. Reactive forms of NO play a critical role in the pathogenesis of CHF and other cardiovascular diseases [54,55]. The formation of certain types of ROS and RNS is biochemically coupled with the generation of other reactive species and free radicals. The hydroxyl radical (•OH) is a powerful oxidizing agent, active in electron acceptance, donation, and transfer reactions. It is involved in the oxidative modification of proteins, nucleic acids, and prostaglandins. Hydrogen peroxide (H_2_O_2_) acts as a pro-oxidant and has high diffusibility. It activates transcription factors such as NF-kappa B and APO-1, and regulates the expression of Cyclooxygenase-2 (COX-2) and iNOS. Singlet oxygen (^1^O_2_) and ozone (O_3_) are also strong oxidants; however, their exact role in the pathogenesis of cardiovascular diseases remains unclear [56]. The peroxyl radical (ROO•) exhibits lower oxidative potential compared with the hydroxyl radical but possesses higher diffusibility. It has been implicated in carcinogenesis and myocardial remodeling. The alkoxyl radical (RO•) is highly reactive with lipids, leading to their oxidative modification. It participates in the formation of cytotoxic and cardiotoxic compounds such as trans-nonenals, malondialdehyde, and other products of lipid peroxidation [57,58]. The hypochlorite anion (OCl^−^) is a potent oxidizing agent, with greater diffusibility than peroxynitrite (ONOO^−^). It participates in the oxidation of sulfonic and disulfonic groups in proteins and DNA, as well as in the chlorination of tyrosine residues. OCl^−^ disrupts multiple signaling pathways, initiates apoptosis, receptor desensitization, and affects transcriptional processes [59].

### 3.3. Pathways of ROS Generation in Heart Failure

In cardiovascular diseases, the activation of ROS production may be attributed to the following mechanisms [60]: discoordination of mitochondrial electron transport chains against the background of increased expression of NAD(P)H oxidase, decreased pO_2_ in the myocardium, and accumulation of reduced forms of pyridine nucleotides; accumulation of catecholamines, their precursors, and metabolic products; activation of adenine nucleotide metabolism and increased expression of xanthine oxidase; imbalance of trace elements in the myocardium (Fe, Cu, Zn, Mn, etc.); enhanced metabolism of arachidonic acid; decreased expression of antioxidant enzymes (superoxide dismutase, catalase, glutathione peroxidase, glutathione reductase); and deficiency of endogenous antioxidants (α-tocopherol, glutathione, cysteine, etc.).

**Figure 3:**
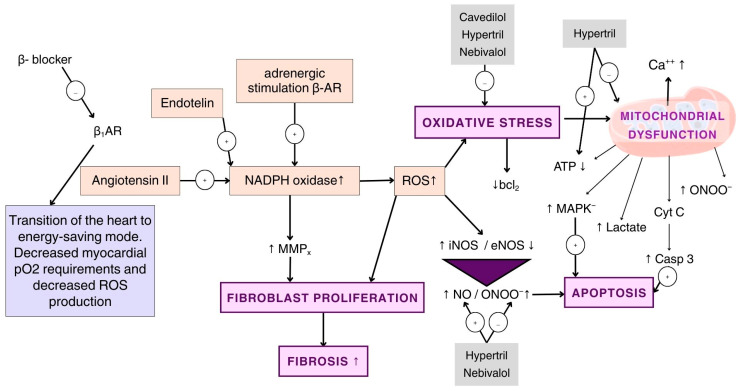
The ROS–NO–Mitochondrial Dysfunction Pathway in the Development of CHF.

A significant increase in the activity and expression of NOX4 in myocardial mitochondria has been observed both in patients with CHF and in experimental animals with various models of CHF. It has been reported that mitochondrial-localized NOX4 in cardiomyocytes mediates mitochondrial dysfunction and decreases mitochondrial DNA (mtDNA) content in the heart during pathological hypertrophy caused by pressure overload and CHF, through the induction of oxidative damage to mitochondrial respiratory complexes and mtDNA [61]. It is also known that NOX4, localized perinuclearly in the endoplasmic reticulum of cardiomyocytes, protects them from pressure overload by promoting myocardial angiogenesis and enhancing the mitochondrial energetic response [62]. These data indicate that ROS produced within cardiomyocytes are important components of an adaptive and coordinated signaling pathway when the heart faces overload stress. However, excessive ROS production during ischemia and arterial hypertension leads to mitochondrial bioenergetic impairment (Figure 3), which hinders the heart’s adaptation to chronic stress and contributes to the development of CHF [63].

Current views on the pathogenesis of CHF indicate that the expression of mitochondrial NOX4 in cardiomyocytes, in response to decreased pO_2_, is a primary source of superoxide radical production. NOX4 plays a key role in initiating and mediating mitochondrial dysfunction, cardiomyocyte apoptosis, suppression of transcription, and ultimately, left ventricular dysfunction and CHF development [64,65]. Moreover, fibrosis, collagen deposition, and activation of metalloproteinases involved in myocardial remodeling during CHF depend on the amount of ROS produced by NOX4. NOX4 participates in the phenotypic transformation of fibroblasts into myofibroblasts, which is associated with the progression of end-stage heart failure [66]. ROS generated by NADPH oxidase, the mitochondrial electron transport chain, and pro-inflammatory cytokines activate mitogen-activated protein kinases (MAPKs) and transcription factors. These, in turn, modulate ion channel functions and ultimately enhance neuronal activity and sympathetic outflow in cardiovascular diseases, particularly in angiotensin II-dependent CHF [67].

Studies indicate that xanthine oxidase is an important source of ROS in cardiovascular diseases, particularly in CHF. ROS produced by xanthine oxidase during the conversion of xanthine to uric acid, as part of adenine nucleotide degradation under ischemic conditions, lead to impaired endothelium-dependent vasodilation, reduced cardiac contractility, and disease progression [68]. ROS are generated in the cyclooxygenase pathway as byproducts at the peroxidase site during prostaglandin synthesis. COX-2 converts arachidonic acid into prostaglandin PgG_2_, which undergoes peroxide oxidation to prostaglandin PgX_2_. ROS are produced during this two-step enzymatic process [69]. In CHF, COX-2 expression is induced and upregulated in the myocardium in response to inflammatory cytokines and hypoxia via the nuclear factor-κB (NF-κB) signaling pathway. Increased COX-2 activity leads to the production of prostaglandins, which play a complex role in the progression of CHF, as well as the generation of ROS. It is hypothesized that COX-2 activation in CHF is associated with left ventricular hypertrophy and the activation of apoptosis [70,71].

Transition metals (Fe, Cu, Zn, Mn) also contribute to ROS generation in the myocardium through redox reactions, particularly via their variable oxidation states. These d-block elements participate in biological processes such as the electron transport chain and enzymatic catalysis, which generate ROS, including superoxide radicals and hydrogen peroxide [72,73]. Among all d-block elements, iron is the most well-known for its role in ROS production. Iron plays a central role in the Fenton and Haber–Weiss reactions—key chemical processes that generate the most cytotoxic ROS, namely hydroxyl radicals. In CHF, a paradoxical situation is observed: despite iron deficiency, the Fenton reaction is activated, leading to increased production of hydroxyl radicals [74]. Iron metabolism in CHF is highly complex, and the mechanisms regulating both systemic and cellular iron homeostasis in cardiovascular diseases remain insufficiently elucidated. Notably, even subphysiological concentrations of iron, under conditions of low pH, elevated hydrogen peroxide levels, and compromised antioxidant defense, may initiate the Fenton reaction [75]. It is also known that Cu^2+^ effectively induces Fenton-like reactions and promotes hydroxyl radical formation [76]. Other sources of ROS in mitochondria are the enzymes monoamine oxidase A (MAO-A) and B, both located in the outer mitochondrial membrane. They catalyze the oxidative deamination of neurotransmitters and biogenic amines, leading to the formation of superoxide radical, singlet oxygen, and hydrogen peroxide [77]. In cardiomyocytes, MAO-A is localized in the outer mitochondrial membrane and contributes to the production of ROS, leading to the accumulation of autophagosomes, mitochondrial fusion, the formation of a conjugate of microtubule-associated protein 1 light chain 3-phosphatidylethanolamine (LC3-PE), and the accumulation of the autophagy receptor p62 and ubiquitinated proteins. The superoxide radical, singlet oxygen, and hydrogen peroxide generated by MAO-A can directly affect mitochondrial function and inhibit sphingosine kinase, resulting in ceramide accumulation and, consequently, cardiomyocyte apoptosis [78].

### 3.4. Antioxidant System of the Myocardium in CHF

The myocardial antioxidant system plays an important role in maintaining various components of energy metabolism, catecholamine metabolism, and prostaglandin synthesis, as well as in regulating the physiological levels of ROS required for proper cell signaling. A reduction in individual components of this system, or its overall activity, leads to uncontrolled ROS production and the development of oxidative stress [79].

It has been noted that in CHF, myocardial activity of mitochondrial Mn-SOD and extracellular Fe-SOD is markedly reduced, contributing to uncontrolled ROS generation through various metabolic pathways. This decline enhances oxidative stress, leading to further ventricular dysfunction, remodeling, and worsening of disease prognosis. A decrease in Fe-SOD mRNA and protein expression has been shown to correlate with the severity of CHF. The expression level of Fe-SOD serves as an informative marker for both CHF prognosis and the potential for endogenous myocardial cytoprotection. Reduced Fe-SOD expression in heart failure has been associated with increased oxidative stress in the myocardium and endothelial dysfunction. Moreover, total SOD activity and that of its isoenzymes are useful predictors of adverse outcomes in patients with CHF and reduced ejection fraction due to dilated cardiomyopathy. Total SOD, Mn-SOD, and Cu/Zn-SOD provide moderate improvement in risk stratification when used in combination with traditional biomarkers and enhance prognostic assessment [80,81]. Catalase plays a crucial role in protecting the myocardium from oxidative stress by decomposing hydrogen peroxide. A low catalase level has been associated with an increased risk of mortality in severe heart failure. A decrease in catalase levels in the coronary sinus correlates with elevated oxidative stress products and one-year mortality in patients with severe CHF [82].

CHF is characterized by decreased GPx4 activity and activation of oxidative stress, which leads to excessive production of free lipid radicals, including lipid aldehydes capable of irreversibly modifying critical cellular components of cardiomyocytes. These self-propagating free lipid radicals can disrupt various metabolic and signaling pathways, resulting in irreversible structural and functional alterations in the myocardium and contributing to the progression of CHF. GPx4 has demonstrated high prognostic value for predicting atrial fibrosis recurrence in CHF and shows a negative correlation with transforming growth factor β, indicating a possible role of ferroptosis in the development of atrial fibrosis [83,84]. The endogenous antioxidant α-tocopherol is capable of protecting fatty acids and membrane phospholipids by scavenging peroxyl radicals and terminating the free-radical chain reaction. Recent studies have shown that low plasma concentrations of α-tocopherol directly correlate with increased mortality and elevated levels of oxidative stress markers in patients with cardiovascular diseases, including CHF [85]. Thus, the reduction in myocardial antioxidant defense in CHF is not only a cause of increased ROS production and oxidative stress development but also contributes to the initiation of ferroptosis, inflammation, and fibrosis.

### 3.5. Oxidative Stress and CHF

Oxidative stress, defined as excessive production of ROS against a background of diminished antioxidant defense and the formation of cyto- and cardiotoxic products of oxidative modification of proteins, lipids, and nucleic acids, is considered a key factor in the pathogenesis of heart failure [86]. Oxidative stress plays a crucial role in the pathophysiology of cardiac remodeling and heart failure. It induces alterations in intracellular pathways and redox signaling, leading to cellular dysfunction and damage to the functional activity of the target organ, the heart. These effects exacerbate maladaptive remodeling and contribute to the progression of CHF [87]. Oxidative stress also promotes myocardial tissue inflammation in CHF, contributing to an increased risk of comorbid conditions such as obesity, diabetes mellitus, and sleep apnea [88,89]. For example, oxidative stress induced by hypertrophic stimuli leads to the oxidation of cysteine SH residues in class II histone deacetylases, which are major negative regulators of cardiac hypertrophy. This event results in the translocation of oxidized products into the cytosol and disruption of transcriptional processes involving NF-κB and the nuclear factor of activated T-cells. Oxidized thiols negatively affect the enhancer factor II—a short DNA region that, by interacting with other transcription factors, promotes gene or gene cluster transcription from core promoters. Oxidative suppression of the enhancer factor in cardiomyocytes contributes to cardiac hypertrophy in CHF [90]. A significant negative correlation has been found between malondialdehyde levels and left ventricular ejection fraction in patients with CHF. It is also known that the levels of lipid peroxides and 8-isoprostanes, the main biochemical markers of oxidative stress, are elevated in the plasma and pericardial fluid of CHF patients and positively correlate with disease severity [91].

Stable products of oxidative stress and ROS stimulate tyrosine kinase Src, the GTP-binding protein Ras, protein kinase C, MAPKs, and c-Jun N-terminal kinase, thereby inducing apoptosis, an important contributor to remodeling and dysfunction. Apoptosis is triggered by ROS and oxidative stress-mediated damage to DNA and mitochondria, as well as by the activation of pro-apoptotic signaling kinases. ROS and oxidative stress products (such as aldehydes, ketones, and lipid peroxides) cause DNA strand breaks, activating the nuclear enzyme poly(ADP-ribose) polymerase 1 (PARP-1). PARP-1 regulates the expression of various inflammatory mediators that promote the progression of cardiac remodeling. ROS can also activate matrix metalloproteinases (MMPs), a family of proteolytic enzymes. MMPs are usually secreted in an inactive form and are post-translationally activated by ROS through interactions with critical cysteine residues in the propeptide autoinhibitory domain. In addition, ROS stimulate the transcription of NF-κB and activator protein-1, which upregulate MMP expression. Increased MMP activity has been demonstrated in heart failure, accompanied by elevated markers of oxidative stress.

### 3.6. NO and Apoptosis in CHF

Cardiomyocyte apoptosis makes a significant contribution to the loss of cardiac tissue and function, ultimately leading to CHF. Various factors, including oxidative stress, impaired NO signaling, inflammation, and mitochondrial dysfunction, can trigger cardiomyocyte apoptosis in CHF. The ultimate consequence of increased cardiomyocyte apoptosis is not only a reduction in the heart’s contractile capacity but also the promotion of adverse remodeling, fibrosis, and, ultimately, increased morbidity and mortality associated with end-stage heart failure [92,93]. The mitochondrial pathway of apoptosis is primarily mediated by the intrinsic signaling cascade, which involves the release of pro-apoptotic factors from mitochondria. The key players in this pathway are members of the Bax family and NO, both of which regulate mitochondrial membrane permeability. When cells experience stress or damage, pro-apoptotic proteins promote the release of cytochrome c from mitochondria into the cytosol. This release activates caspases, particularly caspase 9, which subsequently triggers effector caspases such as caspase 3, ultimately leading to cellular degradation and death [94,95]. In addition, ROS and reactive nitrogen species (RNS) can further amplify apoptotic signaling and promote cell death [96]. It has been shown that NO modulators can inhibit apoptosis by affecting caspase-3 expression or by enhancing the expression of the heat shock protein HSP70 [38,97].

At physiological concentrations, NO produced through endogenous or pharmacological mechanisms can interrupt IL-1β–dependent apoptotic pathways, whereas NO overproduction by inducible synthases or pharmacological donors enhances apoptosis through the formation of peroxynitrite (ONOO^−^) [98]. Measures aimed at preserving NO, reducing its conversion by ROS into peroxynitrite (ONOO^−^), and enhancing the activity of SOD and glutathione-dependent enzymes can inhibit the initiating mechanisms of apoptosis. Oxidative stress, hypoxia, and ischemia, which reduce NO bioavailability and increase the formation of peroxynitrite, nitrosonium ions, and protein nitrosylation, amplify pro-apoptotic signaling transmitted via Fas receptors. Additionally, activation of nitrosative stress and the presence of cytotoxic concentrations of NO in the cell lead to a decrease in the mitochondrial anti-apoptotic protein Bcl-2 and induction of c-Fos. High concentrations of NO can also affect SH-dependent Fe^2+^ regeneration mechanisms and enhance ferroptosis. H_2_O_2_, whose excess is observed under reduced expression of SOD, catalase, and GPx in CHF, contributes to the amplification of NO-dependent apoptotic mechanisms [99]. Similarly, ascorbic acid can enhance the pro-oxidant properties of iron and promote ferroptosis under pH alterations. Low catalase levels contribute to the amplification of H_2_O_2_-induced apoptosis [100].

Macrophages expressing inducible iNOS, characterized by condensed nuclei and cytoplasm, play a specific role in initiating NO-dependent apoptotic mechanisms. NO released by activated macrophages leads to their functional suppression and, ultimately, apoptosis. Several studies using S-nitrosothiols have demonstrated the role of transnitrosylation reactions in apoptosis. These reactions mediate the transfer of active NO species to the SH groups of low-molecular-weight thiols (such as glutathione and cysteine) and signaling proteins, altering or suppressing their function and thereby initiating apoptosis [101,102]. Based on experimental studies, it can be suggested that an increase in reactive nitrogen species, even under conditions of low NO production, leads to inhibition of mitochondrial oxidative phosphorylation through nitrosylation of electron transport chain enzymes and translocator proteins of the outer mitochondrial membrane. Reactive nitrogen species are also capable of inducing phospholipid peroxidation and oxidation of thiol groups present on mitochondrial membrane proteins. Altogether, these processes ultimately result in the massive release of pro-apoptotic factors from mitochondria [103,104]. The release of mitochondrial pro-apoptotic proteins, including cytochrome c, and the subsequent activation of caspases in CHF lead to cellular dysfunction, apoptosis, and contribute to systolic impairment. The concept of “seeing hope in death” refers to the idea that, although apoptosis contributes to heart failure progression, this process can be interrupted in many cardiomyocytes, creating “zombie myocytes” that retain nuclei but suffer from cytoplasmic damage. These cells, though dysfunctional, may retain the potential for recovery through cytoplasmic reconstruction. Pharmacological interventions aimed at inhibiting NO-dependent mechanisms of apoptosis could therefore represent a novel therapeutic approach for CHF [105].

## 4. Modern Perspectives on Pharmacological Therapy of CHF: Focus on β-Adrenergic Blockers

Strategies for the prevention and treatment of CHF have evolved significantly over the past two decades [106]. The stages of CHF have been redefined to include the concept of pre-heart failure (pre-HF), which encompasses asymptomatic patients who have developed structural or functional cardiac abnormalities or exhibit elevated plasma levels of natriuretic peptides or cardiac troponins [107]. First-line therapy for patients with CHF with reduced ejection fraction (HFrEF) includes foundational treatment with angiotensin-converting enzyme (ACE) inhibitors or angiotensin receptor blockers (ARBs), β-blockers, mineralocorticoid receptor antagonists, sodium–glucose co-transporter 2 (SGLT2) inhibitors, and diuretics. For patients with heart failure with mildly reduced ejection fraction (HFmrEF) or preserved ejection fraction (HFpEF), first-line therapy typically includes SGLT2 inhibitors, β-blockers, and diuretics [108].

### 4.1. Sodium–Glucose Co-Transporter 2 (SGLT2) Inhibitors

Over the past decade, SGLT2 inhibitors have been used in patients with type 2 diabetes mellitus (T2DM) to control blood glucose levels. However, among antihyperglycemic agents, only SGLT2 inhibitors (empagliflozin, canagliflozin, dapagliflozin) have demonstrated a positive cardiovascular prognosis. In a subsequent study, only empagliflozin was shown to reduce both all-cause and cardiovascular mortality [109]. Therefore, SGLT2 inhibitors have recently emerged as a new class of drugs with proven efficacy in the treatment of CHF [110]. Studies investigating the mechanisms of SGLT2 inhibitors have shown that their cardioprotective effects are not related to glucose-lowering actions. Evidence indicates that SGLT2 inhibitors exert positive hemodynamic effects, including reductions in blood pressure, cardiac preload and afterload, and vascular resistance, as well as decreased sympathetic activity and left ventricular mass (LVM). According to several researchers, this mechanism largely accounts for the beneficial hemodynamic effects of SGLT2 inhibitors [111]. Another mechanism underlying the cardioprotective effects of SGLT2 inhibitors is their ability to modulate the activity of membrane Na^+^/H^+^ exchangers (NHE) isoforms 1 and 3, whose expression is increased in CHF and T2DM [112]. SGLT2 inhibitors lower glucose and insulin levels, increase glucagon concentrations, and enhance the production of the ketone β-hydroxybutyrate and its conversion to acetoacetate. They also promote the utilization of ketones and free fatty acids as energy substrates, increasing compensatory energy production, which may improve systolic function and myocardial remodeling [113]. However, SGLT2 inhibitors activate a relatively inefficient ATP-generating pathway, namely the utilization of ketones and free fatty acids. Considering that SGLT2 is not expressed in the human myocardium and that data are lacking on the effects of SGLT2 inhibitors on myocardial substrate utilization, mitochondrial function, and the phosphotransfer system in patients with CHF, the impact of these drugs on myocardial energy metabolism remains largely unknown [114]. The cardioprotective effect of empagliflozin in a CHF model was also reflected in the reduced expression of COX-2 and interleukin-1β (IL-1β), independent of T2DM [115]. SGLT2 inhibitors may enhance NO production and improve endothelial function by promoting eNOS phosphorylation (activation) and reducing ROS generation in diabetic rats. They increase NO levels by preventing its interaction with superoxide radicals. However, data on these effects remain very limited [116]. Alongside ACE inhibitors and β-blockers, SGLT2 inhibitors constitute a scientifically validated foundational therapy for patients with HFrEF. A recent meta-analysis involving 95,444 participants across 75 studies demonstrated that the combination of ACE inhibitors, β-blockers, and SGLT2 inhibitors was most effective in reducing all-cause mortality and the composite outcome of cardiovascular death or first hospitalization for HFrEF. However, data on the efficacy of SGLT2 inhibitors for cardiovascular outcomes in patients with CHF with HFpEF remain lacking [117]. However, the impact of SGLT2 inhibitors on myocardial metabolism in CHF remains unclear and continues to be an open question for researchers.

### 4.2. Diuretics

Loop diuretics are the most commonly used diuretics due to their rapid onset of action and high efficacy. They reversibly inhibit the Na^+^/2Cl^−^/K^+^ cotransporter in the thick ascending limb of the loop of Henle, where approximately one-third of filtered sodium is reabsorbed. This inhibition reduces sodium and chloride reabsorption and increases diuresis. Loop diuretics also enhance prostaglandin synthesis, which causes renal and venous vasodilation. This mechanism explains some cardiac effects, such as reduced pulmonary artery wedge pressure. Common loop diuretics include furosemide, bumetanide, torasemide, and ethacrynic acid [118,119].

Thiazide diuretics inhibit the sodium-chloride transporter in the distal segment of the ascending limb of the nephron and the early portion of the distal convoluted tubule. They limit maximal urine dilution, thereby increasing free water clearance and promoting sodium and chloride excretion through the renal tubular epithelium. Thiazides also reduce peripheral vascular resistance via a mechanism that is not yet fully understood, resulting in lowered arterial blood pressure [120].

Potassium-sparing diuretics used in the treatment of CHF include the aldosterone receptor antagonists spironolactone and eplerenone. They act on the cortical segment of the collecting ducts, reducing sodium and water reabsorption while increasing the excretion of hydrogen and potassium ions, with their effects mediated by antagonism of mineralocorticoids. They also significantly reduce the harmful effects of aldosterone on the cardiovascular system. Spironolactone is a non-selective aldosterone receptor antagonist, which can result in endocrine-related side effects, such as gynecomastia.

While there are three main classes of diuretics (loop diuretics, thiazide diuretics including metolazone, and potassium-sparing diuretics), loop diuretics are most commonly used due to their potent natriuretic effect. At the same time, despite their relatively modest diuretic effect, potassium-sparing diuretics have been shown to significantly improve long-term outcomes in patients with symptomatic CHF. Diuretics may negatively affect myocardial metabolism, potentially altering glucose and lipid levels, enhancing cardiac remodeling, and adversely influencing the NO system [118].

### 4.3. ACE Inhibitors

ACE inhibitors are a cornerstone in the treatment of heart failure, particularly HFrEF. They improve cardiac function and survival by reducing angiotensin II levels, which leads to vasodilation (reducing afterload) and decreased aldosterone production (reducing fluid retention and preload) [121].

Compounds containing a sulfhydryl group, such as captopril (Capoten), exhibit a stronger vasodilatory effect. In most patients who have suffered a myocardial infarction, as well as in those with CHF, long-term treatment with captopril has been shown to reduce mortality [122]. ACE inhibitors exhibit anti-ischemic effects to a greater or lesser extent, both those containing roups, such as captopril (Capoten), sistopril, and tenziomin, and those that do not, including enalapril (Renitec, Vasotec, Enap, Enam, Ednit), cilazapril (Inhibace), quinapril (Accupro, Accupril), perindopril (Prestarium, Coversyl), ramipril (Tritace, Altace, Delix), spirapril (Repress), trandolapril (Gopten, Odrik), and fosinopril (Fozinorm, Moex). It is also important to consider the indications for the use of drugs from other pharmacological groups in the treatment of patients with ischemic heart disease. ACE inhibitors improve the course of CHF by reducing afterload, preload, and systolic wall tension, which leads to an increase in cardiac output without raising heart rate [123]. Since the 1980s, several large prospective randomized placebo-controlled trials have demonstrated that ACE inhibitor therapy reduces overall mortality, particularly in patients with heart failure with reduced ejection fraction [22,50,124].

According to clinical trial data, the combination of candesartan with ACE inhibitors, in addition to standard heart failure therapy, leads to a further reduction in cardiovascular mortality in patients with CHF. In this setting, careful monitoring of renal function and serum potassium levels is required [125]. The study results indicate that valsartan is as effective as captopril in patients who have experienced acute myocardial infarction with heart failure and/or left ventricular systolic dysfunction and can be used as an alternative treatment in patients intolerant to ACE inhibitors [126]. Despite these findings, ACE inhibitors remain the first-line therapy for patients who have experienced myocardial infarction, while ARBs may be used in cases of proven intolerance. Although the use of ACE inhibitors may appear beneficial in patients with heart failure and preserved left ventricular systolic function, there are currently no large-scale clinical trials confirming this hypothesis. Evidence suggests that ACE inhibitors can influence NO levels both directly and indirectly—by preventing the formation of angiotensin II (which reduces NO production) and by inhibiting bradykinin degradation (which stimulates local NO release). An increase in NO levels within the microvasculature of coronary arteries and the myocardium contributes to vasodilation, decreased myocardial oxygen demand, modulation of contractility, and improved left ventricular function. Conversely, a reduction in NO production caused by certain ACE inhibitors may exacerbate endothelial dysfunction. These findings highlight the need for further investigation into the NO-related effects of different agents within this pharmacological class [127]. New therapeutic perspectives are emerging with the use of ACE inhibitors. Pharmacological modulation of angiotensin II receptor activity may trigger signaling pathways that promote regeneration of damaged endothelium and prevent vascular thrombosis in heart failure [128]. It has also been shown that pharmacological modulation of the angiotensin II receptor plays a promotive role in the differentiation of bone marrow mesenchymal stem cells into keratinocytes and enhances reparative regeneration processes [129]. It is known that ACE inhibitors promote the expression of ACE2, which mediates the entry of SARS-CoV-2 into cells, causing COVID-19 [130]. Therefore, concerns have arisen regarding the safety of patients receiving ACE inhibitor therapy. However, studies have shown that angiotensin II is a potent activator of the enzyme ADAM17, a protease that cleaves ACE2. Once ACE2 binds to the viral spike protein, it facilitates viral entry into the cell. Thus, inhibition of angiotensin II production by ACE inhibitors may actually reduce the likelihood of infection [131].

### 4.4. β-Adrenergic Blockers

Over the past decade, CHF has evolved from being a contraindication to a direct indication for the use of β-adrenergic blockers. It is well established that activation of the sympathetic-adrenal system lies at the core of the cardiovascular continuum in HF, playing, along with the renin–angiotensin system, a key role at all stages—from the impact on risk factors to the development of overt CHF. From this perspective, the use of β-blockers is entirely logical. The modern rationale for β-blocker therapy in CHF is based on nearly 30 years of experimental and clinical pilot studies and is strongly supported by the results of well-known multicenter trials. By the late 1990s, β-blockers had become a mainstay in the treatment of CHF. Alongside ACE inhibitors, β-blockers are considered first-line agents in HF therapy because they improve survival rates, reduce hospitalizations, increase ejection fraction (EF), and decrease both the mass and sphericity of the left ventricle. β-Blockers exert their cardioprotective effects primarily by reducing heart rate, myocardial contractility, and oxygen demand of cardiomyocytes [132].

Currently, three generations of β-adrenergic blockers are distinguished according to their pharmacological properties. First-generation β-blockers are non-selective, blocking both β_1_- and β_2_-receptors (e.g., propranolol [anaprilin], nadolol, timolol). These agents are associated with significant side effects, which considerably limit their clinical use. Second-generation β-blockers are cardioselective, as they selectively block β_1_-receptors (e.g., metoprolol, bisoprolol, atenolol). They produce fewer adverse effects compared with first-generation agents and demonstrate better long-term tolerability and a strong evidence base for improving survival in patients with CHF. Third-generation β-blockers may be either highly selective for β_1_-receptors (e.g., nebivolol, betaxolol) or non-selective (e.g., carvedilol, labetalol, carteolol). These drugs possess additional anti-ischemic, endotheliotropic, antioxidant, antiproliferative, antihypertrophic, and antiapoptotic properties, with minimal side effects. β-blockers of the first and second generations are often referred to as traditional, whereas those of the third generation are known as β-blockers with additional pharmacological properties [133,134].

The mechanism of action of this group of drugs is associated with the attenuation of sympathetic influences on the myocardium and a reduction in myocardial oxygen demand. The most widely used agent in clinical practice is anaprilin (propranolol), which exhibits antianginal, antihypertensive, and antiarrhythmic properties, although these effects become pronounced only at higher doses. This phenomenon is attributed to the membrane-stabilizing effect of propranolol. Propranolol suppresses the activity of the sinoatrial node and ectopic foci of excitation, increases the effective refractory period of the atrioventricular node, decreases the automaticity of cardiac cells, inhibits conduction through the atrioventricular node, and reduces myocardial excitability. Under the influence of propranolol, a decrease in overall cardiac workload is also observed. In addition, the drug exhibits a mild sedative effect. In cases of acute myocardial infarction, propranolol has been shown to reduce mortality and improve quality of life [135].

Among the side effects of non-selective β-adrenoblockers are bronchospasm, hyperglycemia, atrioventricular block, increased peripheral vascular tone, physical fatigue, decompensation of heart failure, hypertriglyceridemia, hyperglycemia, insulin resistance, reduced exercise tolerance, impotence, and decreased cognitive function of the central nervous system [136,137,138]. Selective β-blockers such as atenolol, bisoprolol, metoprolol, and nebivolol do not cause bronchospasm, peripheral blood stasis, or alterations in glucose tolerance. Among the cardioselective β1-blockers, atenolol, bisoprolol, and metoprolol succinate remain the reference drugs for the treatment of CHF in many countries. In recent years, nebivolol has been widely used [139,140].

Data on the effects of bisoprolol on the NO system are limited and contradictory. There is some evidence that bisoprolol exhibits endothelial-protective activity and increases NO production in diabetes. However, the potency of this effect is significantly weaker than that of nebivalol. There is also evidence that bisoprolol lacks this effect. A study of bisoprolol in patients with HFrEF involving 2647 participants demonstrated a significant 34% reduction in mortality [141]. Metoprolol is a selective β1-blocker characterized by a shorter duration of action compared with atenolol and bisoprolol. In the Metoprolol in Dilated Cardiomyopathy (MDC) study, which included 383 patients with HFrEF, treatment with metoprolol resulted in a 34% reduction in the relative risk of all-cause mortality [142]. Nebivolol is a selective β1-receptor antagonist that also stimulates β3-receptors, leading to NO production and vasodilation. Nebivolol is currently the only well-studied β-blocker with NO-mimetic activity. Experimentally, nebivolol modulates eNOS expression, leading to excess NO without causing oxidative stress. In animal models, nebivolol normalizes the eNOS/iNOS ratio, limits oxidative stress during cardiac ischemia/repudiation, stimulates endothelial adenosine triphosphate production, increases endothelial calcium levels via P2Y receptors, and determines calcium-dependent activation of eNOS. Nebivolol is able to inhibit ROS formation by mitochondrial energy-producing systems in experiments [143]. In the study “Study of the Effects of Nebivolol Intervention on Outcomes and Rehospitalization in Seniors with Heart Failure,” which included more than 2000 patients over the age of 70 with reduced ejection fraction, a significant reduction was observed in the combined endpoint of all-cause mortality and hospitalizations related to CHF [144]. Among third-generation adrenergic blockers with additional peripheral vasodilating properties, “hybrid” adrenergic blockers are distinguished—such as labetalol, celiprolol, dilevalol, carvedilol, bucindolol, nebivolol, altioprim, and cardiotril. These agents are used in the treatment of angina pectoris, ischemic heart disease, hypertension, and CHF [134,145]. Currently, carvedilol is the most widely used agent—a moderately selective β-blocker without intrinsic sympathomimetic activity that also exhibits peripheral vasodilatory properties due to α-adrenergic blockade. By blocking cardiac β-adrenergic receptors, carvedilol can lower blood pressure and reduce heart rate, while not decreasing cardiac output. Carvedilol inhibits the renin–angiotensin–aldosterone system by blocking renal β-adrenergic receptors, resulting in reduced plasma renin activity. Through α1-adrenergic blockade, the drug induces peripheral vasodilation, thereby decreasing systemic vascular resistance. Experimentally, carvedilol was able to increase eNOS expression during cadmium cardiotoxicity and normalize the eNOS/iNOS ratio during cardiac ischemia/reperfusion. The experiment also found that carvedilol increased NO production by influencing P2Y purine receptors on glomerular endothelial cells [143].

Carvedilol has been successfully used in the treatment of arterial hypertension, CHF, and stable angina pectoris. In the US Carvedilol Heart Failure Study (which included 1094 patients with HFrEF), the use of carvedilol was associated with a 27% reduction in the risk of hospitalization due to cardiovascular causes and a 38% reduction in the combined risk of hospitalization or death [146]. Hemodynamic studies in patients with CHF have demonstrated that carvedilol possesses a safer profile compared with other agents, particularly in terms of cardiac index and systemic vascular resistance [147]. Beta-blockers exert an anti-apoptotic effect in heart failure by reducing harmful chronic adrenergic stimulation of the heart, which can lead to cardiomyocyte death. By blocking these toxic effects, beta-blockers protect cardiomyocytes from apoptosis and necrosis, thereby slowing the detrimental process of left ventricular remodeling, improving cardiac function, and ultimately reducing mortality in patients with CHF [147,148]. Treatment with β-blockers such as metoprolol, carvedilol, and bisoprolol inhibits oxidative stress reactions while improving the clinical course of CHF. The antioxidant effects of β-blockers have not been sufficiently studied and require further investigation; however, several mechanisms have been proposed. First, the β-blocking effect is important, since catecholamines induce the production of ROS in the myocardium. Second, the anti-ischemic and negative chronotropic effects also play a significant role. Third, β-blockers are believed to possess scavenging properties that depend on their chemical structure [149,150].

Thus, β-adrenergic blockers are indicated for CHF of any severity and have secured a firm position among first-line therapies for this condition. However, current HF treatment guidelines do not provide differentiated schemes for the use of β-blockers based on their affinity for various β-adrenergic receptor subtypes, or on their effects on myocardial and systemic metabolism. Unresolved issues include the differential application of β-blockers across diverse HF patient categories and the early detection of resistance to these drugs. It also remains unclear in which clinical scenarios a particular β-blocker would be most appropriate. These gaps underscore the need for further studies on the efficacy of β-blockers in HF, as well as the development of novel molecules exhibiting β-adrenergic blocking activity with additional properties, such as antioxidant, metabolotropic, NO-mimetic, and anti-apoptotic effects [151].

### 4.5. Medicines of Different Groups Used for Heart Failure

Vericiguat is methyl (4,6-diamino-2-(5-fluoro-1-(2-fluorobenzyl)-1H-pyrazolo [3,4-b]pyridin-3-yl)pyrimidin-5-yl) carbamate, a stimulator of soluble guanylate cyclase (sGC). As we wrote above, modulation of the biological effects of NO looks quite attractive from the point of view of preventing the occurrence and progression of endothelial dysfunction, which is considered as the most important mechanism of CHF development. And considering that the bioavailability of NO in CHF is significantly reduced due to oxidative stress and antioxidant deficiency, the use of donors and precursors of NO synthesis in the form of nitrates, derivatives of sydnone imine, and L-arginine, which do not address the main causes of decreased NO bioavailability, is characterized by low efficiency. An alternative approach is the modulation of the activity of NO-dependent salt-soluble guanylate cyclase. The progression of CHF is associated with decreased soluble guanylate cyclase activity, decreased tissue perfusion, and increased peripheral vascular resistance, accompanied by a deficiency of circulating NO and oxidative stress. Vericiguat actively restores the NO-sGC-cGMP signaling pathway by directly stimulating sGC independently and synergistically with NO. This increases intracellular cGMP levels, which can improve myocardial and vascular function. Vericiguat has been FDA-approved since 2021. Clinical trials of vericiguat in patients with CHF and an ejection fraction of less than 45% have shown some positive results, although further research is required [24,152]. Serelaxin is a recombinant analog of the endogenous human peptide relaxin 2, which belongs to the RXFP1 (Relaxin Family Peptide Receptor 1) family of relaxins, which exert a direct vasodilatory effect through stimulation of membrane-associated G-protein-coupled receptors. Serelaxin activates signaling pathways (AMPK-AKT), leading to eNOS expression, as well as a number of slow-type signaling pathways (ALK 5/Smad2/3), promoting stimulation of the endothelin type B receptor and the expression of proangiogenic growth factors. Serelaxin has confirmed its ability to significantly reduce the severity of dyspnea in patients with acute heart failure; however, the drug did not affect mortality rates or the need for rehospitalizations in the short term. However, the use of serelaxin resulted in a significant decrease in the plasma levels of sodium uretic peptide, troponin T, and creatinine in patients with acute heart failure, a decrease in pulmonary vascular pressure, and an increase in daily diuresis [153,154,155]. All this requires additional research. In the 1970s, direct renin inhibitors were developed: enalkiren, remikiren, and zankiren, which were studied in patients with arterial hypertension. However, these drugs had low bioavailability and unsuitable pharmacokinetic parameters when administered orally. The emergence of a new representative of direct renin inhibitors—aliskiren—a drug for oral administration significantly increased interest in this group of drugs. Aliskiren inhibits the binding of renin to specific receptors and inhibits the formation of angiotensin II through renin-dependent and renin-independent mechanisms. Aliskiren prevents the “escape” phenomenon characteristic of other modulators of the renin-angiotensin system—ACE inhibitors and angiotensin receptor blockers. Clinical trials showed that aliskiren, compared with placebo, contributed to a small reduction in the relative risk of cardiovascular death and rehospitalization due to CHF. Clinical trials of aliskiren were stopped due to an increased incidence of hyperkalemia, hypotension, and end-stage renal disease, and a lack of significant clinical benefit [156,157,158,159,160,161]. Omecamtiv mecarbil, a low-molecular-weight cardiac myosin activator, is a promising drug with significant inotropic potential for the treatment of CHF. Compared with β1-stimulants (milrinone, vesnarinone, and dobutamine), it does not increase calcium influx from the sarcoplasmic reticulum. It has been experimentally established that by binding to the catalytic domain of cardiomyocyte myosin ATPase, omecamtiv mecarbil increases the rate of myosin transition to the actin-bound state, leading to an increase in the contact time of the actomyosin complex and an increase in stroke and minute volumes while reducing heart rate [162,163,164]. Clinical studies have shown that the use of omecamtiv mecarbil in patients with CHF with reduced ejection fraction resulted in an increase in stroke volume, left ventricular ejection fraction, and a decrease in heart rate, without affecting blood pressure. Omecamtiv mecarbil has also demonstrated efficacy and safety in the treatment of CHF with reduced ejection fraction and low systolic pressure. However, omecamtiv mecarbil’s effectiveness in reducing cardiovascular mortality has been somewhat disappointing for its developers and clinicians [165]. In a pooled analysis of the effects of all doses used, the effect of omecamtiv mecarbil on dyspnea was not different from that of placebo. Results from recent clinical trials of omecamtiv mecarbil in CHF with reduced ejection fraction (~27–28%) did not show significant reductions in overall mortality or improvements in exercise capacity and did not reveal any significant benefit in terms of overall functional status [166]. Thus, among the drugs developed for the treatment of CHF with a new mechanism of action, omecamtiva mecarbil and serelaxin proved successful at the initial stage of research. Large clinical trials will show how effective they really are in the treatment of CHF.

### 4.6. 1,2,4-Triazole Derivatives as Promising Agents for Cardioprotection in Heart Failure

1,2,4-Triazole derivatives exhibit a broad spectrum of pharmacological activities, including anti-hypoxic, anti-ischemic, antioxidant, growth-stimulating, antiviral, and anti-inflammatory effects [167,168]. 1,2,4-Triazole derivatives are of particular interest as potential cardioprotective agents. Among 3-methyl-1,2,4-triazolyl-5-carboxylic acids and their salts, several compounds have demonstrated cardioprotective activity in rat models of myocardial infarction. Within this subgroup, notable salts include morpholine, sodium, and monoethanolamine salts of 3-methyl-1,2,4-triazolyl-5-thioacetic acid, with the most prominent representative being thiotriazoline. Thiotriazoline, the first member of the class of metabolotropic cyto- and cardioprotectors, exhibits anti-ischemic, cardioprotective, antioxidant, energotropic, membrane-protective, and immunomodulatory properties. The results of preclinical studies on animal models are presented in the review [169,170,171].

Results from multicenter randomized studies support the inclusion of thiotriazoline in the comprehensive treatment of cardiovascular diseases as a metabolotropic cardioprotector. Thiotriazoline reduces the frequency and duration of ischemic episodes and cardiac rhythm disturbances, increases tolerance to physical exertion, and improves both the quality and longevity of life in patients with stable angina, myocardial infarction, post-infarction myocardial remodeling, and CHF, thereby enhancing prognosis and patient quality of life. Thiotriazoline has demonstrated good tolerability and safety when administered in a daily dose of 600 mg over an 8-week course for the treatment of ischemic heart disease, stable angina (functional class II–III), and CHF (8298 patients in total). Clinical trials have shown a low incidence of adverse effects, approximately 3%, which were mild to moderate in severity and not directly attributable to the drug. The proven effects of thiotriazoline include a significant reduction in the incidence of ventricular arrhythmias and correction of rhythm disturbances, as well as a beneficial effect on quality of life, as described in the review [172]. The mechanism of action of thiotriazoline, established in experimental studies on animal models of myocardial ischemia, involves increasing the expression of antioxidant enzymes, reducing the concentration of free radicals, activating the compensatory malate–aspartate energy shuttle, normalizing the function of the Krebs cycle, and initiating redox-dependent expression of transcription factors under ischemic conditions [173,174]. Further modification of the thiotriazoline molecule led to the creation of a new compound with the following structure: L-lysine 3-methyl-1,2,4-triazolyl-5-thioacetate, named Angiolin. Angiolin increases the bioavailability of NO, normalizes the expression of eNOS and iNOS mRNA, and increases the density of myocardial vascular endothelial cells and VEGF expression in the myocardium of rats and rabbits during the moderation of experimental myocardial infarction, myocardial ischemia, and CHF in rats and rabbits. Angiolin normalizes systolic blood pressure, reduces the manifestations of ischemic dysfunction of the left ventricle—increases left ventricular pressure, increases working and stroke volumes, and reduces total peripheral vascular resistance. Angiolin improves myocardial energy metabolism by activating the compensatory malate-aspartate shunt of ATP formation during myocardial ischemia [175,176,177]. Cardioprotective properties were also identified in 4-amino-1,2,4-triazole derivatives [178]. Among them, the most active identified compound was 1-(β-phenylethyl)-4-amino-1,2,4-triazolium bromide (working name Hypertril), a cardioselective beta-blocker with NO-mimetic effect, demonstrating antihypertensive, antianginal, antiischemic, fibrinolytic, and antioxidant properties [179]. Administration of Hypertril to rats with experimental myocardial infarction and CHF resulted in normalization of ECG and cardiohemodynamic parameters—a reduction in systolic and diastolic myocardial dysfunction and restoration of autonomic mechanisms of heart rate regulation. This also reduced cardiac preload by decreasing heart rate, while increasing stroke volume. It also reduced cardiac afterload by decreasing total vascular resistance. Administration of Hypertril to rats with experimental CHF resulted in a significant increase in myocardial NOS activity in the cytosol and mitochondria due to an increase in eNOS (increased eNOS-positive cell density and increased eNOS expression), and also contributed to the normalization of myocardial iNOS expression. A course of intragastric administration of Hypertril to animals with experimental CHF reduces the manifestations of secondary mitochondrial dysfunction and normalizes myocardial energy metabolism—an increase in the charge of the inner mitochondrial membrane and an increase in ATP levels in the cytosol and mitochondria. The potential drug Hypertril exhibits a pronounced antihypertensive and cardioprotective effect after 15 days of administration in SHR rats with stable arterial hypertension, manifested by a significant decrease in blood pressure and a reduction in target organ damage in the heart. It also mitigates disturbances in the L-arginine-NOS-NO system in the myocardium in arterial hypertension. Hypertril exhibits NO-mimetic properties, enhancing NO synthesis by increasing the expression of endothelial NOS in the myocardium. Administration of Hypertril to hypertensive rats reduces cardiomyocyte nuclear density to levels observed in normotensive rats [32,180,181]. Based on comprehensive experimental studies and analysis of data on disturbances in cardiac electrical activity, autonomic regulation of heart rate, oxidative stress activation, impaired energy metabolism and ATP production, as well as dysfunction of the nitric oxide system, we obtained new insights into the role of altered expression of iNOS and eNOS mRNA in the pathogenesis of CHF. These studies demonstrated that the severity of disturbances in cardiac electrical activity and autonomic regulation, oxidative stress, and energy deficit are associated with dysfunction of the myocardial NO system. Our experimental studies have shown some advantage (in terms of the effect on some molecular, biochemical, immunohistochemical, and electrophysiological parameters) of Hypertril over traditional beta-blockers of different generations (metoprolol, carvedilol, bisoprolol, and nebivolol) in experimental CHF [182,183,184].

The potential of Hypertril as a treatment for cardiovascular diseases has been confirmed by other studies [185]. It should be emphasized that the identified properties of the cardioprotective action of new molecules of 1,2,4-triazole derivatives aimed at interrupting the interconnected NO-dependent mechanisms of cardiac destruction in heart failure are consistent with the data of the modern literature demonstrating the key role of disturbances in cellular metabolism, oxidative stress, the nitroxidergic system, mitochondrial dysfunction, immune response and endothelial dysfunction in the pathogenesis of a wide range of cardiovascular diseases [186]. The pharmacological activity of 1,2,4-triazole derivatives, in particular their antioxidant, anti-inflammatory, cardioprotective, beta-blocking, and cytoprotective properties, has been demonstrated in experimental studies [186], which further confirms the possibility of using 1,2,4-triazole as a basis for the construction of new potential drugs. Furthermore, genetic and transcriptional features described in studies of cardiovascular, endocrine, and immune-inflammatory diseases [187] highlight the key role of molecular abnormalities in the development of pathology, providing a scientific basis for the development of new approaches to pathogenetic therapy [188,189]. Thus, our results on the experimental evaluation of the efficacy of new potential drugs and the study of their mechanism of action fit organically into the modern scientific context and confirm the prospects for further study of these drugs as means of targeted action on key links in cardiac dysfunction.

## 5. Study Limitations

### Several Key Limitations Should Be Noted

Incomplete Pathway Analysis: The review did not comprehensively analyze all potential NO-related pathways involved in the pathogenesis of CHF. The study did not analyze the role of NO in initiating ferroptosis in CHF, the role of NO in the mechanisms of inflammatory responses in CHF, or the role of NO in immunological activation. The study did not analyze cardiac fibrosis pathways, autophagy, epigenetic regulation, or the cardiorenal-metabolic axis. The review did not analyze the duration of the cardioprotective effects of basic medications, and the potential for sustained benefits remains unexplored.

Unexplored Sex Differences: The review did not analyze potential sex differences in the formation of NO-dependent mechanisms of cardiac destruction in CHF or potential sex differences in drug response, limiting the generalizability of the results.

## 6. Translational and Clinical Significance of the Study

The most important results of the study for clinical medicine should be noted. There is the possibility of using the following as additional diagnostic biomarkers in a laboratory diagnosis of CHF: Markers of mitochondrial dysfunction, namely circulating mtDNA, which significantly increases in the blood with mitochondrial damage —specifically, impaired integrity of the mitochondrial outer membrane and opening of the mitochondrial permeability pore (mPTP), cytochrome C, lactate/pyruvate ratio, manganese-dependent superoxide dismutase (intramitochondrial); a change in its activity indicates mitochondrial damage, NAD(P)H oxidase, the activity of which is associated with mitochondrial dysfunction; markers of the nitroxidergic system, including a decrease in the level of stable NO metabolites (NOx) and an increase in the level of nitrosative stress markers (nitrotyrosine) may be potential diagnostic and prognostic indicators in heart failure and indicate a decrease in NO bioavailability. A decrease in reduced glutathione and a decrease in glutathione peroxidase activity may also indicate a decrease in NO bioavailability. Reduced eNOS expression and a decrease in the eNOS/iNOS ratio may also be potential diagnostic markers of CHF. Impaired NO metabolism is a key pathogenetic marker in many cases of heart failure with preserved ejection fraction. These markers can help differentiate chronic heart failure and predict its severity, along with standard diagnostic markers.

Theoretical rationale for the inclusion in complex therapy of CHF: pharmacological agents for the correction of mitochondrial dysfunction and energy metabolism (direct mitoprotectors, activators of cytosolic-mitochondrial shunts, macroergic agents); pharmacological agents for the correction of the nitric oxide system (positive modulators of eNOS expression, NO synthesis precursors, agents that increase NO bioavailability and protect it from reactive oxygen species).

Theoretical justification for the development and creation of new original agents combining the properties of beta-blockers and NO-mimetics, antioxidants, and myoprotectors. The promise of the results presented in the review is confirmed by the fact that the new potential drugs Angiolin (as a metabolite-tropic cardio- and endothelioprotector) and Hypertril (as a beta-blocker and NO-mimetic) have been approved for clinical trials and phase 1 trials and have been admitted to phase 2 trials.

## 7. Conclusions

Recently, national cardiology societies have developed guidelines for the treatment of CHF with reduced left ventricular ejection fraction. However, recommendations for the drug treatment of CHF with mid- and preserved left ventricular ejection fractions are being refined and require additional research. Basic approaches to CHF therapy consider diuretics, vasodilators, and inotropic agents as initial symptomatic therapy. These agents are used as a temporary measure to reduce fatigue, dyspnea, edema, fluid retention, and achieve hemodynamic stability. However, these agents have virtually no impact on patient survival or readmission rates. As the study results presented in the review demonstrate, increased survival, reduced readmissions, and improved clinical outcomes occur with the inclusion of appropriately selected ACE inhibitors, angiotensin receptor blockers, and, especially, beta-blockers in the treatment regimen. The presented material on the mechanisms of myocardial injury in CHF demonstrates the complexity of their analysis, interactions, and the order of initiation. The emergence of new experimental and clinical data shows that these mechanisms are much broader, more diverse, and more complex than previously understood. However, given a number of substantiated promising target links in the progression of CHF (mitochondrial dysfunction, apoptosis, impaired absorption of energy metabolism substrates, dysfunction in the NO system), as well as taking into account comorbid conditions of various types of CHF (diabetes mellitus, chronic kidney disease, obesity, hyperuricemia, hypothyroidism, arterial hypertension, atrial fibrillation, COPD), which worsen the prognosis, increase the number of hospitalizations and mortality and determine the need to search for new ways of effective treatment. Based on this, new principles in such areas of CHF treatment include mitoprotection, NO modulation, improvement of myocardial energy metabolism, antioxidant modulation, anti-inflammatory action, membrane protection, and anti-apoptotic action. These can improve survival, reduce the risk of non-fatal clinical outcomes, and enhance the quality of life and clinical status of patients with CHF. This can be achieved through the development of new original drugs, such as beta-blockers with additional properties (antioxidant, NO-modulating, anti-apoptotic, mitoprotective, and anti-inflammatory), as well as by evaluating these properties in standard therapy drugs or combining them with metabolite-modifying agents. The results presented and summarized in this review contribute to a broader understanding of the NO-related molecular and biochemical mechanisms underlying the development of myocardial dystrophy in CHF, as well as the effects of ACE inhibitors, diuretics, and β-blockers of various generations, and the novel drug Hypertril, used in the treatment of cardiovascular diseases, on energy metabolism and the nitric oxide system. These insights may aid in the rational selection of these agents for inclusion in comprehensive CHF therapy.

## Data Availability

The paper contains the original contributions made during the study; further inquiries can be directed to the corresponding authors.
